# Action observation combined with gait training to improve gait and cognition in elderly with mild cognitive impairment A randomized controlled trial

**DOI:** 10.1590/1980-57642020dn14-020004

**Published:** 2020

**Authors:** Rommanee Rojasavastera, Sunee Bovonsunthonchai, Vimonwan Hiengkaew, Vorapun Senanarong

**Affiliations:** 1 Faculty of Physical Therapy, Mahidol University, Nakhon Pathom, Thailand.; 2 Gait and Balance Group, Faculty of Physical Therapy, Mahidol University, Nakhon Pathom, Thailand.; 3 Division of Neurology, Faculty of Medicine Siriraj Hospital, Mahidol University, Bangkok, Thailand.

**Keywords:** action observation, cognition, gait, gait training, mild cognitive impairment, observação da ação, cognição, treinamento da marcha, marcha, comprometimento cognitivo leve

## Abstract

Owing to advancement of medical technology and current knowledge, the population has a longer life expectancy, leading to an increase in the proportion of elderly.

**Objective:**

The study aimed to investigate the effect of action observation (AO) combined with gait training on gait and cognition in elderly with mild cognitive impairment (MCI).

**Methods:**

Thirty-three participants were randomly allocated to action observation with gait training (AOGT), gait training (GT), and control (CT) groups. The AOGT and GT groups received a program of observation and gait training protocol with the same total duration of 65 min for 12 sessions. For the observation, the AGOT group watched a video of normal gait movement, while the GT group watched an abstract picture and the CT group received no training program. All participants were assessed for gait parameters during single- and dual-tasks using an electronic gait mat system and were assessed for cognitive level using the Montreal Cognitive Assessment (MoCA) at baseline, after training and at 1-month follow-up.

**Results:**

The results showed that the AOGT group had significant improvements in gait speeds during single- and dual-tasks, as well as better MoCA score, while the GT group had significant improvement only in gait speed.

**Conclusion:**

The adjunct treatment of AO with gait training provides greater benefits for both gait and cognitive performances in elderly with MCI.

## Resumo.

Com o avanço da tecnologia médica e do conhecimento atual, a população tem uma expectativa de vida mais longa, levando a um aumento na proporção de idosos.

**Objetivo:**

O estudo teve como objetivo investigar o efeito da observação de ação (AO) combinada com o treinamento da marcha na marcha e cognição em idosos com comprometimento cognitivo leve (CCL).

**Métodos:**

Trinta e três participantes foram alocados aleatoriamente para observação de ação com grupos de treinamento de marcha (AOGT), treinamento de marcha (GT) e controle (CT). Os grupos AOGT e GT receberam um programa de observação e protocolo de treinamento de marcha com a mesma duração total de 65 minutos por 12 sessões. Na observação, o grupo AGOT assistiu a um vídeo de movimento normal da marcha, enquanto o grupo GT assistiu a uma figura abstrata e o grupo CT não recebeu nenhum programa de treinamento. Todos os participantes foram avaliados quanto aos parâmetros da marcha durante tarefas simples e duplas, utilizando um sistema eletrônico de esteira da marcha e avaliados quanto ao nível cognitivo, utilizando a Avaliação Cognitiva de Montreal (MoCA) na linha de base, após o treinamento e 1 mês de acompanhamento.

**Resultados:**

Os resultados mostraram que o grupo AOGT apresentou melhorias significativas nas velocidades da marcha durante tarefas simples e duplas, além do escore MoCA, enquanto o grupo GT teve melhora significativa apenas na velocidade da marcha.

**Conclusão:**

O tratamento adjunto da AO com o treinamento da marcha proporciona maiores benefícios tanto do desempenho da marcha quanto do desempenho cognitivo em idosos com CCL.

Owing to advancement of medical technology and current knowledge, the population has a longer life expectancy, leading to an increase in the proportion of elderly. Aging is characterized by degenerative change in musculoskeletal, cardiopulmonary, neurological and cognitive systems. Regarding cognitive function, mild cognitive impairment (MCI) is a pre-dementia phase where one or more cognitive domains declines, interfering with the performance of more complex tasks in daily life. However, memory and other cognitive functions may be restored by adjusting medication or resolving the exact causes.^1^ In addition, a longitudinal study by Shimada et al. in 2019^2^ suggested that specific lifestyle activities, such as driving, using a map to travel to unfamiliar places, reading books or newspapers, and other forms of participative activities may play significant roles for MCI reversion. Besides the noticeable decline in cognitive ability, gait abnormality can also manifest in the MCI stage. It usually presents with decreased gait speed and increased gait variability, especially during testing under attention-demanding, challenging tasks.^3^

It is well known that early treatment can reduce the likelihood of developing severe symptoms, hospitalization and financial burden.^4^ Treating MCI is crucial because it can develop to more severe stages such as dementia. However, pharmacological treatment for this group has not proven completely successful and side effects have been observed.^5^ Therefore, nonpharmacological treatment becomes of more interest. The American Academy of Neurology guidelines in 2018,^5^ recommended that exercise has a benefit for MCI on general health and cognition. However, discovering the most appropriate exercise program is key for more specific and effective results in this population.

Neurodegenerative cognitive decline was found to be linked to the integrity of the mirror neuron system (MNS). Comparisons of functional magnetic resonance imaging when observing movement and real executing of functions, indicated the activation of the fronto-parietal network in the classical MNS and the superior temporal gyrus areas for normal elderly. Although a lesser extent of the parietal area was activated, the superior temporal gyrus area was not activated for MCI, and neither of these areas were activated for Alzheimer’s disease (AD).^6^ The MNS is the neural circuit that plays a role in intentional understanding, empathy, self-recognition, action imitation and the evolution of language.^7^ Hence, decline in the MNS may affect the ability to regulate the early planning phase and mirroring process.

Scherder et al.^8^ proposed a strategy on the basis of “last in and first out” principle for gait rehabilitation in dementia. It refers to the principle of neural circuits that mature early and are less vulnerable to deterioration. This may provide preventive and rehabilitative strategies for higher-level gait disturbances concerning different methods. Some vulnerable neuronal circuits of patients with early dementia were related to the loss of function in gait control, alternative foot placement and movement inhibition. These functions are under the control of MNS, which is able to be activated when individuals perform motor imagery (MI), action observation (AO), and real execution. In recent rehabilitative programs, motor functional training together with observation or imagination was suggested to facilitate MNS activation and enhance motor performance.^9^^-^ 11 AO refers to an observational practice that has been used extensively for the goal of motor programming and enhances motor learning and performance. MI is a dynamic state allowing learners to simulate motor actions mentally, without actual execution.^11^ Both approaches are safe, low cost, adaptable and consume little time, creating the potential benefit for a rehabilitation training program in conjunction with the other types of conventional motor training.^9^ However, MI practice may have more limitations for individuals with cognitive decline and provides lower neuron network activation when compared with AO.^11^ In AO, motor-related information can be available through the visual function by encoding into the mental representation of the memory to organize the intended action.^12^ However, MI requires a conscious effort to retrieve a stored mental representation of the memory in order to form.^13^

Currently, AO and MI have become emerging rehabilitation strategies to enhance motor and cognitive functions in different neurological conditions.^9^^,^ 14 ^-^ 17 Cuenca-Martinez et al. in 2020^18^ summarized the roles of MI and AO in the motor learning process. They explained that movement representation in the brain and cortical-subcortical networks related to planning, executing, adjusting and automatic real executing, share a similar neurophysiological activity. This activity underlying the creation and consolidation of motor representation may differ in terms of level and function of MNS activation between AO and MI. The MNS appears to function more efficiently in motor learning, but AO is less cognitive demanding than MI. However, cognitive ability can be gained more through observing complicated tasks or using cues in patients with Parkinson’s disease.^15^^,^ 19

Given the points outlined, AO might be more suitable for use in the elderly with MCI. Therefore, the study aimed to investigate the effect of AO combined with gait training on gait and cognition in elderly with MCI. We hypothesized that there would be a greater improvement of gait and cognition in the action observation with gait training (AOGT) group than in the gait training (GT) and control (CT) groups.

## METHODS

### Study design and ethics consideration

This study design was a three-arm randomized controlled trial using single-blind treatment allocation for participants. Before participating in the study, all subjects received the research details and provided their informed consent approved by the Central Institutional Review Board of Mahidol University (MU-CIRB COA no: 2018/081.1004).

### Sample size estimation

The sample size was estimated based on a previous study^20^ investigating the effect of multicomponent exercise on gait in the elderly with amnestic MCI. Their results showed a mean and standard deviation for gait speeds at baseline and after the intervention of 1.10±0.32 and 1.38±0.32 m/s, respectively, calculated using the equation $\left[n={\left(Z_{1-\alpha /2}+Z_{1-\beta }\right)}^{2}\sigma^{2}/\delta^{2}\right]$. The alpha error was set at 0.05 and power was set at 0.80, and the estimated sample number was 10 participants per group. Thus, the final number of 11 participants per each group in the present study was considered sufficient.

### Participants

Participants with amnestic MCI were recruited from the Memory Clinic of Siriraj hospital and communities surrounding Phuttamonthon District, Thailand. They were diagnosed as having MCI according to the core clinical criteria of the National Institute on Aging and the Alzheimer’s Association.^1^ The criteria included alteration in cognition relative to previous functioning, impairments in at least one of the cognitive domains that are greater than expected for the individual’s age and education, preserved ability to perform activities of daily living, and no dementia. Subjects were included in the study that met the following criteria: aged 60-80 years, exhibiting memory deficit, scoring 18-24 on the Montreal Cognitive Assessment (MoCA), fully independent for activities of daily living, as measured by the Barthel Index, being able to walk independently without the use of gait aid, having gait speed during dual-task<1 m/s, and proving able to follow the instructions. Participants were excluded if they had any disease that affected gait performance, had moderate-to-severe severity levels of depression, defined by a score>9 on the Patient Health Questionnaire-9 (PHQ-9), diagnosis of dementia by a neurologist, dizziness or headache on the assessment day, and unable to correct vision or hearing impairments by use of glasses or hearing aid.

**Figure 1:**
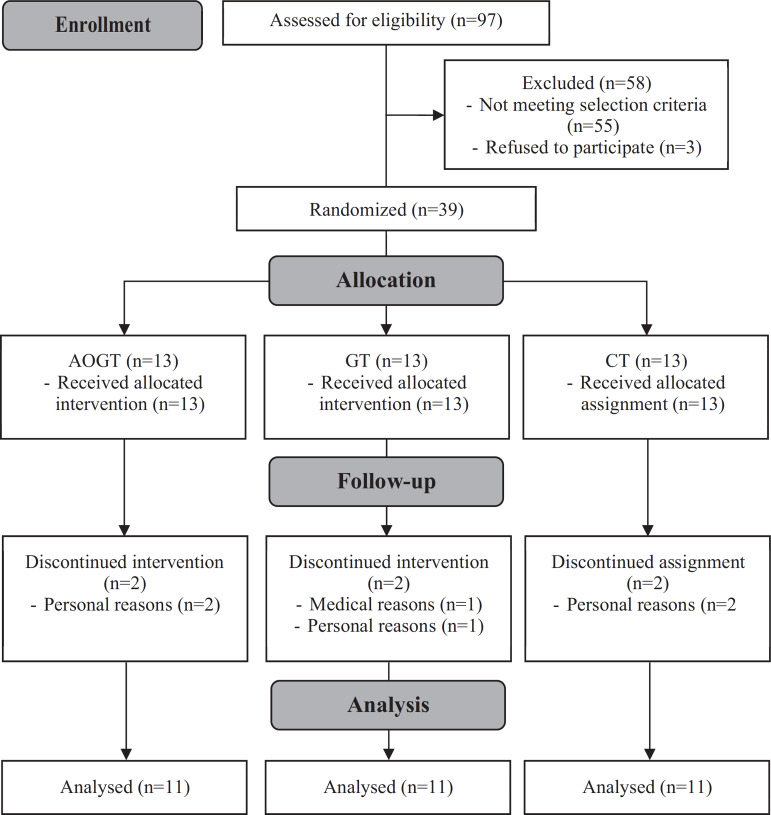
Study protocol.

As shown in Figure 1, 97 elderly were initially screened using the selection criteria. Subsequently, 58 participants were excluded for not meeting the selection criteria or due to difficulties enrolling on the study, such as a long distance from home to the assessment venue and/or duration of participation. Therefore, the remaining 39 subjects were randomized into each assigned group (AOGT, GT, and CT). All participants were stratified by age (60-70 or 70-80 years) and by number of years of education (<6 or>6 years). The researcher then randomly selected lottery numbers that were placed in sealed envelopes at a ratio of 1:1:1 to allocate the three groups of participants. During the study, five participants dropped out due to personal reasons (n=5) and one for medical reasons (n=1). Thus, a final total of 33 participants completed the whole study protocol.

### Procedures

The study was conducted between April 2018 and August 2019 at the Faculty of Physical Therapy, Mahidol University, Nakhon Pathom, Thailand. Participants of the AOGT and GT groups were assessed for outcomes at three timepoint [baseline (T1), after training (T2), and at 1-month follow-up (T3)]. In addition, the CT group was also assessed for the same outcomes at the same timepoints as the intervention participants.

### Outcome measures

The primary outcomes comprised gait variables, which were collected using the electronic gait mat system (Zebris force distribution measurement platform, size 307 × 60.5 × 2.1 cm [L × W × H], S/N: 1243020-0015-0816, Allgäu Region, Germany). This system was proven to be valid and reliable equipment for measuring gait variables, and is usually used in both clinical and research settings.^21^^,^ 22 Gait data were captured at a sampling rate of 100 Hz and WinFDM Software, Version 0.1.11 was used to extract the spatiotemporal gait variables. Averaged gait speed (m/s), stride time variability (%CoV) and stride length variability (%CoV) from nine trials in each of the single- and dual-tasks were used for further analysis. The co-efficient of variation (CoV) was the method used to measure the variability of gait variables, calculated as (standard deviation/mean) * 100.^23^

The secondary outcome comprised global cognition, assessed using the Thai version of the MoCA.^24^ The MoCA is a multi-domain cognitive test having a possible total score of 30 with higher scores indicating better performance. The Thai-MoCA was assessed for internal consistency and criterion validity using the Clinical Dementia Rating Scale. Good internal consistency was found for the instrument, with a Cronbach’s alpha coefficient of 0.744. When cut-off scores under 25 and 18 were set for MCI and AD, a sensitivity and specificity of 0.70 and 0.95, and of 0.80 and 0.95 were found, respectively.

### Intervention

The training programs for the AOGT and GT groups are shown in Table 1. Participants in the AOGT and GT groups received the training program from an experienced physiotherapist for 12 sessions with 2 or 3 sessions weekly. Both groups received the same amount of training with a total time of 65 min per session. Training sessions comprised observation (5 min), warm-up (5 min), gait training (40 min), cool-down (5 min), and stretching (10 min), consecutively. For the observation section, the AOGT group watched a video of walking acted out by a normal healthy individual. The actor walked at the speed of 120 beats/min identified by a metronome, and stepped on markers placed on the floor 60 cm apart for each step. The GT group watched abstract pictures of Vincent van Gogh to reduce the influence of emotions. After completing the 12 training sessions, participants performed gait training by themselves at home for three sessions weekly over a 1-month period. To monitor participant compliance, they were requested to note the training in a logbook and were followed up by telephone weekly. The CT group received no training program, but was educated about dementia on the screening day and lived as usual for two months. None of the participants reported any risk or harm as a result of this study.

**Table 1:** Training programs for action observation with gait training (AOGT) and gait training (GT) groups.

Observation training (5 min)	Warm-up (5 min)	Gait training (40 min)	Cool-down (5 min)	Stretching (10 min)
**AOGT group:** The video shows the model who walks normally with a metronome and steps on the markers placed on the floor **GT group:** The video shows Van Gogh's paintings	1) Swinging leg forward and backward 2) Swinging leg from side to side 3) Kicking leg 4) Standing on tiptoes *All exercises performed 20 times per side	Sessions 1-3: Gait with markers and metronome 100 steps/min Sessions 4-6: Gait with markers and metronome 120 steps/min Sessions 7-9: Gait with metronome 120 steps/min Sessions 10-12: Independent gait	1) Swinging leg forward and backward 2) Swinging leg from side to side 3) Kicking leg 4) Standing on tiptoes *All exercises performed 20 times per side	1) Sitting on chair with legs extended and reaching toward toes 2) Sitting on chair and placing hands behind buttocks. Keeping back straight and squeezing shoulder blades together 3) Sitting on chair and grasping knee to chest 4) Standing with legs wide apart. Leaning toward one leg and bending the knee until opposite leg feels tight *All exercises held for 20 sec and performed 3 times per side

### Statistical analysis

SPSS, Version 24 (SPSS, Inc., IBM Company, Chicago, IL, USA) was used for data analysis, with the statistical significance set at p<0.05. The Kolmogorov-Smirnov Goodness of Fit Test was used to determine whether the data had a normal distribution. Two-way mixed ANOVA was used to evaluate the effects of group (AOGT, GT, and CT), time (T1, T2, and T3) and interaction effect of group by time on the variables assessed. For further analysis, repeated measure ANOVA with LSD post-hoc test was used to compare means of variables at each assessment timepoint for each group. In addition, one-way ANOVA with LSD post hoc test was used to compare change in variables between baseline and post-training (T2-T1) and between baseline and 1-month follow-up (T3-T1) among the three groups of participants.

## RESULTS

The demographic characteristics of the participants are presented in Table 2. The data showed no differences (p>0.05) in the data among these three groups.

**Table 2:** Characteristics of participants in action observation with gait training (AOGT), gait training (GT) and control (CT) groups.

Characteristics		AOGT (n=11)	GT (n=11)	CT (n=11)	p-value
Age (years)^a^		67.64±4.64	67.50±5.60	65.71±2.45	0.530
Sex (Male:Female)^b^		2:9	3:8	3:8	0.852
Education (years)^a^		13.50±3.25	13.82±4.35	12.73±4.43	0.811
Body Mass Index (kg/m^2^)^a^		24.08±3.19	24.71±3.69	25.38±3.80	0.697
Underlying disease^b^	Diabetes Mellitus	0	4	1	0.051
	Hypertension	6	5	5	0.889
	Hyperlipidemia	4	2	3	0.641
Lifestyle (active:sedentary)^b^		5:6	6:5	7:4	0.701
PHQ-9 (score)^a^		2.82±2.82	2.27±2.45	1.91±1.92	0.680
Short FES-I (score)^a^		11.27±5.52	11.36±3.72	8.82±1.40	0.242
Sensation in LE (intact)^b^		11	11	11	1.000
Chair stand test (number)^a^		14.82±4.94	14.82±2.93	16.00±4.29	0.744
Single-leg stand test (s)^a^	Eyes open	38.67±22.72	39.05±21.23	44.93±21.09	0.752
	Eyes closed	6.70±6.84	4.73±4.38	9.69±12.88	0.422
Duration for 12 sessions of training (week)^c^		4.91±0.83	4.55±0.82	-	0.314
Borg scale after gait training phase (score)^c^		11.55±2.42	11.64±2.11	-	0.926

LE: lower extremities; PHQ-9: Patient Health Questionnaire-9; Short FES-I: shortened version of falls efficacy scale. Data expressed as mean±standard deviation or number.

a Significant difference tested by one-way ANOVA for p<0.05;

b Significant difference tested by Kruskal-Wallis H test for p<0.05;

c Significant difference tested by independent *t*-test for p<0.05.

### Main effect and interaction effect for time and group

Significant main effects for time were found in gait speed during single-task (F_1.60,47.85_=20.916, p<0.001), gait speed during dual-task (F_2,60_=23.655, p<0.001) and MoCA score (F_2,60_=31.734, p<0.001), but no significant effect was observed for time in stride time variability during single-task (F_2,60_=0.811, p=0.449), stride time variability during dual-task (F_2,60_=0.503, p=0.608), stride length variability during single-task (F_2,60_=0.840, p=0.437) or stride length variability during dual-task (F_2,60_=1.108, p=0.337).

A significant main effect of group was observed in stride time variability during single-task (F_2,30_=4.059, p=0.028). However, no significant effect of group was found in gait speed during single-task (F_2, 30_=3.004, p=0.065), gait speed during dual-task (F_2,30_=1.740, p=0.193), stride time variability during dual-task (F_2,30_=0.703, p=0.503), stride length variability during single-task (F_2,30_=1.276, p=0.294), stride length variability during dual-task (F_2,30_=2.554, p=0.095) or MoCA score (F_2,30_=0.508, p=0.607).

Significant interaction effects for time and group were observed in gait speed during single-task (F_3.19,47.85_= 7.328, p<0.001) and gait speed during dual-task (F_4, 60_= 6.409, p<0.001). However, no significant interaction effect was found in stride time variability during single-task (F_4,60_=0.263, p=0.901), stride time variability during dual-task (F_4,60_=1.685, p=0.165), stride length variability during single-task (F_4,60_=1.157, p=0.339), stride length variability during dual-task (F_4,60_=2.002, p=0.106) or MoCA score (F_4,60_=2.222, p=0.077).

### Within-group comparison and post-hoc analysis

Table 3 shows within-group comparison and post-hoc analysis of the variables assessed. Significant differences were observed in gait speed during single- and dual-task for both AOGT and GT groups (p<0.05) and in stride length variability during single-task for the AOGT group (p=0.020).

**Table 3:** Within-group and pairwise comparison of variables assessed.

Variables	T1 (mean±SD)	T2 (mean±SD)	T3 (mean±SD)	df (time)	df (error)	F value	p-value^a^	p-value^b^ (T1 vs. T2)	p-value^b^ (T1 vs. T3)	p-value^b^ (T2 vs. T3)
**Gait speed (single-task) (m/s)**										
AOGT	0.86±0.16	1.02±0.15	1.01±0.15	2	20	25.843	<0.001	<0.001	<0.001	0.252
GT	0.94±0.14	1.03±0.10	1.06±0.10	1.305	0.065	9.424	0.006	0.038	<0.001	0.223
CT	0.88±0.15	0.88±0.15	0.87±0.17	2	20	0.235	0.793	-	-	-
**Gait speed (dual-task) (m/s)**										
AOGT	0.69±0.17	0.86±0.22	0.84±0.23	2	20	32.638	<0.001	<0.001	<0.001	0.393
GT	0.70±0.24	0.82±0.24	0.82±0.23	2	20	8.836	0.002	0.009	0.009	0.940
CT	0.64±0.18	0.62±0.19	0.68±0.18	2	20	2.394	0.117	-	-	-
**Stride time variability (single-task) (%CoV)**										
AOGT	2.42±1.15	2.48±0.73	2.33±0.89	2	20	0.065	0.937	-	-	-
GT	1.85±0.78	1.86±0.71	1.80±0.72	2	20	0.030	0.970	-	-	-
CT	2.67±0.98	2.87±1.21	2.33±1.12	1.335	13.354	1.553	0.242	-	-	-
**Stride time variability (dual-task) (%CoV)**										
AOGT	5.30±3.90	3.89±2.29	3.73±2.06	2	20	2.944	0.076	-	-	-
GT	5.34±4.93	4.95±5.95	4.19±4.85	2	20	1.685	0.211	-	-	-
CT	5.41±2.63	6.34±4.40	6.61±3.74	2	20	0.572	0.573	-	-	-
**Stride length variability (single-task) (%CoV)**										
AOGT	1.94±0.41	1.81±0.54	1.64±0.52	2	20	4.824	0.020	0.225	0.017	0.060
GT	1.65±0.50	1.75±0.55	1.85±0.61	2	20	0.461	0.637	-	-	-
CT	2.18±0.88	2.15±0.91	1.87±0.51	2	20	0.901	0.422	-	-	-
**Stride length variability (dual-task) (%CoV)**										
AOGT	2.72±1.79	2.20±0.81	2.01±0.79	1.313	13.128	2.332	0.147	-	-	-
GT	2.92±1.25	2.31±0.72	2.45±0.93	2	20	2.429	0.114	-	-	-
CT	2.90±0.88	3.33±1.35	3.28±1.15	2	20	0.769	0.477	-	-	-
**MoCA (score)**										
AOGT	22.18±1.47	24.64±2.34	26.82±2.04	2	20	30.373	<0.001	0.001	<0.001	0.012
GT	22.64±1.36	24.00±2.86	25.00±3.16	2	20	4.280	0.028	0.124	0.019	0.226
CT	22.18±1.94	24.45±2.91	24.54±2.91	2	20	8.727	0.002	0.003	0.007	0.892

AOGT: Action observation with gait training; GT: gait training; CT: control; T1: baseline; T2: after training; T3: at 1-month follow-up;

a p-values tested by repeated measure ANOVA;

b p-values tested by LSD post-hoc analysis; p-values in bold represent significant differences.

Post-hoc analysis demonstrated significant differences (p<0.05) in gait speed during single- and dual-tasks between T1 and T2 and between T1 and T3 for the AOGT group. The GT group showed a difference in gait speed during single-task between T1 and T2 and between T1 and T3. Regarding stride length variability, a significant difference was found between T1 and T3 for the AOGT group. In addition, significant differences (p<0.05) in MoCA scores were found among assessment timepoints in all groups (AOGT, GT and CT). Post-hoc analysis demonstrated significant differences (p<0.05) between T1 and T2, T1 and T3, and T2 and T3 for the AOGT group, between T1 and T3 for the GT group and between T1 and T2 and between T1 and T3 for the CT group.

### Between-group comparison and post-hoc analysis

Table 4 shows between-group comparison and post-hoc analysis of the variables assessed. To compare the data between groups properly, the values that changed after treatment (T2-T1) and at 1-month follow-up (T3-T1) from baseline (T1) were used in the analysis.

**Table 4:** Between-group and pairwise comparison of variables assessed.

Variables	AOGT (mean±SD)	GT (mean±SD)	CT (mean±SD)	df (Between Groups)	df (Within Groups)	F value	p-value^a^	p-value^b^ (AOGT vs. GT)	p-value^b^ (AOGT vs. CT)	p-value^b^ (GT vs. CT)
**Gait speed (single-task) (m/s)**										
T2-T1	0.17±0.10	0.09±0.12	0.00±0.08	2	30	7.084	0.003	0.085	0.001	0.057
T3-T1	0.15±0.09	0.12±0.08	-0.01±0.10	2	30	10.613	<0.001	0.399	<0.001	0.002
**Gait speed (dual-task) (m/s)**										
T2-T1	0.17±0.07	0.12±0.13	-0.02±0.07	2	30	12.770	<0.001	0.248	<0.001	0.001
T3-T1	0.15±0.08	0.12±0.13	0.04±0.10	2	30	3.619	0.039	0.520	0.015	0.063
**Stride time variability (single-task) (%CoV)**										
T2-T1	0.06±1.49	0.01±0.89	0.20±0.95	2	30	0.079	0.924	-	-	-
T3-T1	-0.09±1.73	-0.05±1.19	-0.34±0.69	2	30	0.158	0.855	-	-	-
**Stride time variability (dual-task) (%CoV)**										
T2-T1	-1.41±2.50	-0.38±2.63	0.93±3.18	2	30	1.941	0.161	-	-	-
T3-T1	-1.57±2.76	-1.15±1.90	1.20±3.55	2	30	3.077	0.061	-	-	-
**Stride length variability (single-task) (%CoV)**										
T2-T1	-0.14±0.35	0.10±0.51	-0.03±0.84	2	30	0.427	0.657	-	-	-
T3-T1	-0.30±0.35	0.20±0.75	-0.31±0.91	2	30	1.861	0.173	-	-	-
**Stride length variability (dual-task) (%CoV)**										
T2-T1	-0.52±1.23	-0.61±0.99	0.43±1.05	2	30	3.020	0.064	-	-	-
T3-T1	-0.70±1.38	-0.46±0.91	0.38±1.16	2	30	2.624	0.089	-	-	-
**MoCA (score)**										
T2-T1	2.45±1.63	1.36±2.69	2.27±1.90	2	30	0.832	0.445	-	-	-
T3-T1	4.64±1.86	2.36±2.80	2.36±2.29	2	30	3.430	0.046	0.031	0.031	1.000

AOGT: Action observation with gait training; GT: gait training; CT: control; T1: baseline; T2: after training; T3: at 1-month follow-up;

a p-values tested by one way ANOVA;

b p-values tested by LSD post-hoc analysis; p-values in bold represent significant differences.

Significant differences (p<0.05) were observed in gait speed during single- and dual-tasks for T2-T1 and T3-T1. Post-hoc analysis revealed significant differences (p<0.05) in gait speed during single-task for T2-T1 between the AOGT and CT groups and in gait speed for T3-T1 both between AOGT and CT groups and between the GT and CT groups. Significant differences (p<0.05) were also found in gait speed during dual-task for T2-T1 both between the AOGT and CT groups and between the GT and CT groups, and in gait speed during dual-task for T3-T1 between the AOGT and CT groups. Concerning the comparison of MoCA scores among the three groups, a significant difference (p<0.05) for T3-T1 was found and post-hoc analysis showed differences (p<0.05) both between the AOGT and GT groups and between the AOGT and CT groups.

## DISCUSSION

This study provided additional information about the beneficial effect of combined AO with gait training on gait and cognition improvement in the elderly with MCI. According to a related review of articles about the effect of AO on motor functions in several conditions, such as Parkinson’s disease, stroke, children with cerebral palsy and post-surgery,^9^^,^ 17 ^,^ 25 evidence was found of using the approach as an adjunctive treatment with routine motor function training to restore upper and lower limb function, as well as postural control. However, very few studies have been conducted in individuals with cognitive decline. The majority of new therapeutic approaches that have emerged are applicable to individuals with cognitive deficits, with the aims of stimulating brain function to restore cognitive and mobility functions.^8^^,^ 26 ^,^ 27 Several pieces of evidence support the relationship of different specific cognitive domains and mobility indicators in older adults, MCI and diseases.^16^^,^ 28 Physical exercise is proposed as a gene modulator to induce structural and functional changes in the brain, promoting benefits for both cognitive and physical function.^27^

Baseline characteristics may have affected the findings of the study, such as age, sex, years of education, whereas others did not differ. Consequently, the results of this study probably indicate the exact effect of each type of training program on the participants. For within-group comparison results, we found the greatest increase in gait speed during the single-task after training when compared with baseline of 0.16 m/s (18.60%) for the AOGT group and 0.09 m/s (9.57%) for the GT group. This increased gait speed during dual-task was also found for the AOGT group of 0.17 m/s (24.64%) and for the GT group of 0.12 m/s (17.14%). This demonstrated the effect of AO in accelerating the motor learning process. The participants were able to learn and improve gait ability by obtaining information concerning action and sequence of movement from the video. Subjects had to memorize and then coordinate their body parts to perform in different situations with appropriate action.^10^ This phenomenon was explained by the mirror theory. During observation of action performed by other people, the superior temporal sulcus receives a visual input of the observed action and sends that information to the mirror neurons to plan the imitative action which comprises the initial phase of motor learning.^7^ To train attention when watching video and gait practice, markers and a metronome were used to help the participants concentrate on their walking.^19^

At 1-month follow-up, both AOGT and GT groups had performed the same gait training by themselves at home. During this period, no AO training was provided for the AOGT group. When comparing gait speed at 1-month follow-up with speed after training, both the AOGT and GT groups exhibited stable gait speeds during both single- and dual-tasks. However, the CT group showed no difference on any of the gait variables for the assessment periods. Therefore, maintaining gait speed, but without improvement during this period, may have been the result of the training program. Adjustment of frequency, duration and intensity should be given greater focus to produce a more efficient performance.^29^ Continuous gait training may retain the function, but has proved less efficient in the elderly because of decline in neuron synapse function in motor memory formation.^30^ In addition, this study tested gait only at a comfortable speed. More challenging conditions, such as at fast speed or gait endurance may be added. In the study of Celnik et al.,^30^ the combination of physical training and AO created a motor memory in the primary motor cortex and modulated motor cortical excitability in agonist and antagonist muscles of the training task, but physical training or AO alone did not. Adjunct AO may facilitate the building of motor memory and motor relearning, which is consistent with the Hebbian theory.^31^ In addition, AO activates visuo-motor interactions through the cognitive process and may improve dual-task or motor-cognitive performances.^11^ For between-group comparison results, no difference was found in gait speed between the AOGT and GT groups. This may have resulted from both groups receiving the same gait training program. However, when compared with the CT group at 1-month follow-up, significant improvements were observed in gait speeds during single- and dual-tasks in the AOGT group, while the GT group showed significant improvement of gait speed during the single-task only.

For other gait variables, stride time variability and stride length variability in both tasks were not improved significantly in any of the groups, except for stride length variability. Significantly reduced stride length variability was found at 1-month follow-up compared with baseline for the AOGT group, with the value of 0.30 %CoV (15.46%). Notably, gait variability during dual-task after training and at 1-month follow-up tended to decrease in the AOGT and GT groups, while increasing in the CT group. Gait variability represents a higher cortical function in the planning process, navigation and sensorimotor integration to control rhythmic stepping. Abnormal cortical function affects the automatic gait movement control system, leading to inconsistent and inaccurate steps.^23^ The present study trained gait timing and foot placement using a metronome and markers placed on the floor from sessions 1 to 9 to practice correct temporo-spatial stepping. These external cues provided feedback for the correct steps and enhanced motor learning. Subsequently, independent gait in training sessions 10 to 12 aimed to transfer the skill to walking in a real situation without markers or metronome. Approximately 9 weeks of training may be too short and unable to show the effect of the intervention on stride time variability. This corresponds with the study of Wang et al.,^32^ which found no change in stride time variability after 8 weeks of physical training, but whose beneficial effect persisted for 12 weeks after training.

In addition, the increase in global cognition, as assessed by the MoCA, promoted significant improvements in scores across all groups, with the highest scores at 1-month follow-up compared with those at baseline (scores of 4.64, 2.36, and 2.36for AOGT, GT, and CT groups, respectively). The increased scores found in the CT and GT groups may relate to the ability of learning and remembering contents of the test.^33^ Another issue may have been due to the active lifestyle of the participants. Preserved physical activity in daily life is a protective factor for cognitive decline through the enhancement of angiogenesis, neurogenesis and the anti-inflammatory environment in the brain.^27^ AO is recognized as one of the cognitive training approaches^9^^,^ 12 that can activate the MNS which is partially damaged in MCI.^6^ A recent study of Caligiore et al.^15^ found improved cognitive domains (working memory and attention) after AO combined with dual-task training for 4 weeks in Parkinson’s disease. They proposed an explanation of cognitive improvement through the mechanism of stimulating goal-setting within the MNS by AO and the mechanisms of working memory and goal maintaining by dual-task training. Improved cognitive ability has also been confirmed by a longitudinal study of Shimada et al.,^2^ who found that this ability can be restored in individuals with MCI who regularly engage in cognitively challenging activities.

Although the sample size was estimated from a previous investigation, the present study may be limited by the low number of participants that satisfied the criteria and completed the overall protocol. Due to limitation by the strict criteria and being controlled among groups of participants, this may affect the application of findings to other kinds of cognitively impaired populations. Hence, a higher number of participants and different types of populations may be needed to obtain greater generalizability. In addition, further studies with more than one blinded factor could be designed, thereby reducing any bias that may exist.

In conclusion, the findings showed that AO combined with gait training was highly beneficial for use in exercise training programs to increase gait speed, reduce stride length variability and improve global cognitive function in the elderly with MCI. The combined effect of AO and gait training was superior to gait training alone, with the latter demonstrating improved gait speed only, when compared to the group without training.
